# Annual report on National Clinical Database 2021 for gastroenterological surgery in Japan

**DOI:** 10.1002/ags3.12868

**Published:** 2024-10-17

**Authors:** Sunao Ito, Arata Takahashi, Hideki Ueno, Shuji Takiguchi, Yoshiki Kajiwara, Yoshihiro Kakeji, Susumu Eguchi, Takanori Goi, Akio Saiura, Akira Sasaki, Hiroya Takeuchi, Chie Tanaka, Masaji Hashimoto, Naoki Hiki, Akihiko Horiguchi, Satoru Matsuda, Tsunekazu Mizushima, Hiroyuki Yamamoto, Yuko Kitagawa, Ken Shirabe

**Affiliations:** ^1^ The Japanese Society of Gastroenterological Surgery Tokyo Japan; ^2^ Department of Gastroenterological Surgery Nagoya City University Graduate School of Medical Sciences Nagoya Aichi Japan; ^3^ Department of Health Policy and Management, School of Medicine Keio University Tokyo Japan; ^4^ Department of Healthcare Quality Assessment Graduate School of Medicine The University of Tokyo Tokyo Japan; ^5^ Department of Surgery National Defense Medical College Tokorozawa Saitama Japan; ^6^ Division of Gastrointestinal Surgery, Department of Surgery Kobe University Graduate School of Medicine Kobe Hyogo Japan; ^7^ Department of Surgery Nagasaki University Graduate School of Biomedical Sciences Nagasaki Japan; ^8^ First Department of Surgery University of Fukui Fukui Japan; ^9^ Department of Hepatobiliary‐Pancreatic Surgery Juntendo University Graduate School of Medicine Tokyo Japan; ^10^ Department of Surgery, School of Medicine Iwate Medical University Yahaba Iwate Japan; ^11^ Department of Surgery Hamamatsu University School of Medicine Hamamatsu Shizuoka Japan; ^12^ Department of Gastroenterological Surgery Nagoya University Graduate School of Medicine Nagoya Aichi Japan; ^13^ Department of Gastroenterological Surgery Toranomon Hospital Tokyo Japan; ^14^ Department of Upper Gastrointestinal Surgery Kitasato University School of Medicine Sagamihara Kanagawa Japan; ^15^ Department of Gastroenterological Surgery, Fujita Health University School of Medicine Bantane Hospital Nagoya Aichi Japan; ^16^ Department of Surgery Keio University School of Medicine Tokyo Japan; ^17^ Department of Gastroenterological Surgery Osaka Police Hospital Osaka Japan; ^18^ Division of Hepatobiliary and Pancreatic Surgery, Department of General Surgery, Graduate School of Medicine Gunma University Maebashi Gunma Japan

**Keywords:** annual report, emergency surgery, gastroenterological surgery, National Clinical Database, surgical outcome

## Abstract

**Aim:**

The Japanese National Clinical Database, which covers more than 95% of the surgeries performed in Japan, is the largest nationwide database. This is the 2021 annual report of the Gastroenterological Section of the National Clinical Database, which aims to present the short‐term outcomes of cases registered in 2021 and discuss significant changes and insights into gastroenterological surgeries observed over the decade.

**Methods:**

We reviewed the data of patients registered in the National Clinical Database between 2012 and 2021.

**Results:**

In total, 5 788 093 cases, including 597 780 cases in 2021, were extracted from the National Clinical Database. The number of surgeries resumed its original trend after a uniform decrease due to the coronavirus disease 2019 pandemic. The patient population continues to age, and the proportion of female patients is steadily increasing. The trend of surgeries being conducted in certified institutions with the involvement of board‐certified surgeons is consistently rising. Moreover, the increasing trend of endoscopic surgery rate is still maintained. Although operative mortality is declining, the trend of increasing postoperative complications continues. Surgery on the esophagus, liver, and pancreas has shown substantial improvements in operative mortality, with a high participation rate of board‐certified surgeons. Surgical procedures with a high incidence of emergency surgeries are characterized by low participation rates of board‐certified surgeons, increased morbidity rates, and worse mortality outcomes.

**Conclusion:**

This overview of surgical patients in Japan, obtained using data extracted from the National Clinical Database, may serve as a critical cornerstone for the future development of gastroenterological surgery.

## Graphical abstract

This study presents the annual report of the gastroenterological surgery of the National Clinical Database (NCD) in 2021. In total, 5 788 093 cases, including 597 780 cases in 2021, were extracted from the NCD. The patient population continues to age, and the proportion of female patients is steadily increasing. The trend of surgeries being conducted in certified institutions with the involvement of board‐certified surgeons is consistently rising.

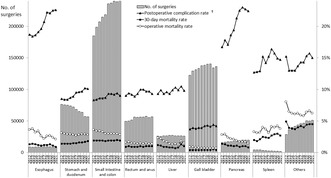

## 1 INTRODUCTION

The Japanese National Clinical Database (NCD) is the largest nationwide database, which covers more than 95% of the surgeries performed in Japan.^1^, ^2^ Data registration with the NCD commenced in 2011 and now includes 1.5 million cases annually from more than 5600 facilities, reaching a cumulative total of 17 million cases.^3^

The Japanese Society of Gastroenterological Surgery (JSGS) is among the 10 societies involved in the establishment of the NCD and conducts numerous systems and analyses using data in the registry to benefit patients, surgeons, and enrolled facilities. First, the registered cases are used for the board certification system; 121 gastroenterological procedures are classified into three levels of difficulty, forming the basis of board certification applications. Second, nine major surgical procedures were benchmarked to improve the quality of surgical care^4^; namely, esophagectomy, partial/total gastrectomy, right hemicolectomy, low anterior resection, hepatectomy, pancreaticoduodenectomy, surgery for acute diffuse peritonitis, and liver transplantation. Risk models for mortality^5^, ^6^, ^7^, ^8^, ^9^, ^10^, ^11^, ^12^, ^13^ and morbidity^13^, ^14^, ^15^, ^16^, ^17^, ^18^, ^19^, ^20^, ^21^ associated with these nine major procedures have been reported as some of the most significant findings derived from NCD. Third, feedback systems based on risk models were constructed and operated online.^4^ For individual patients, a risk calculator can be used to estimate surgical risks based on preoperative clinical information. Furthermore, the NCD can provide surgical outcomes for the enrolled facilities in comparison with national real‐world data. The former can be used to obtain informed consent, whereas the latter can support risk management and performance improvements in facilities.

As mentioned above, the NCD have evolved into an indispensable framework for the advancement of gastroenterological surgery in Japan. The purpose of this report is to understand the current trends and challenges of gastroenterological surgeries and to clarify the path for future advancements, by overviewing gastroenterological surgeries registered in the NCD. Hence, we continue with previous annual reports^22^, ^23^, ^24^, ^25^, ^26^ by presenting the short‐term outcomes of cases registered in the NCD in 2021 and discuss significant changes and insights into gastroenterological surgeries observed over the decade from 2012 to 2021.

## 2 PATIENTS AND METHODS

The included patients were those who underwent one or more of the 121 surgical procedures stipulated in the “Training Curriculum for Board‐Certified Surgeons in Gastroenterology,” and had their surgical data recorded from 2012 to 2021 in the NCD system. The clinical data of these patients were collected using the NCD system, as previously reported.^4^, ^22^, ^23^, ^24^, ^25^, ^26^ The number of surgeries, patient sex, age, postoperative complications, mortalities, and proportions of endoscopic and emergency surgeries for each surgical procedure were calculated. The proportion of institutions certified by the JSGS and the participation rates of anesthesiologists and board‐certified surgeons were calculated. Using these data, four main analyses were conducted: analysis of (1) each surgical procedure; (2) annual trends in surgeries by organ; (3) annual trends of endoscopic surgery rate in major surgical procedures; and (4) surgical procedures with high rates of emergency surgery.

Major requirements for board‐certified training institutions include 600 or more gastroenterological operations, as mandated by the accreditation committee, with at least 120 being essential major surgeries done over the preceding 3 years. Board‐certified surgeons are required to undergo training for over 5 years and have experience in performing more than 450 surgical cases at the aforementioned board‐certified training institutions.

When interpreting the data provided, it is necessary to consider the following factors: (a) due to the NCD's limitation of recording a maximum of eight operative procedures per case, the aggregate number of surgeries reported for each result does not accurately represent the total number of surgical cases; (b) data entries with inaccuracies in patient age, sex, and postoperative 30‐day status were omitted from the analysis; (c) postoperative complications that were grade III or more in the Clavien–Dindo (C‐D) classification were defined as severe complications^27^; (d) the measure of postoperative 30‐day mortality encompasses all instances of death within 30 days following the surgery, irrespective of whether the patient was discharged or not, whereas operative mortality accounts for all deaths occurring during the index hospitalization, which may extend up to 90 days, as well as any deaths occurring after discharge within 30 days from the date of surgery.

## 3 RESULTS

### 3.1 Gastroenterological operative procedures in the “Training Curriculum for Board‐Certified Surgeons in Gastroenterology” in 2021

From January 1, 2021, to December 31, 2021, 597 780 patients underwent gastroenterological surgeries, as recorded in the NCD. In the analyses regarding the treated organ, the breakdown of the cases were as follows: esophagus, 8905 cases (1.5%); stomach and duodenum, 56 759 cases (9.5%); small intestine and colon, 240 448 cases (40.2%); rectum and anus, 56 536 cases (9.5%); liver, 26 250 cases (4.4%); gallbladder, 136 111 cases (22.8%); pancreas, 19 722 cases (3.3%); spleen, 1833 cases (0.3%); and others, 51 216 cases (8.5%).

Characteristics of the operative procedures outlined in the “Training Curriculum for Board‐Certified Surgeons in Gastroenterology” for 2021 are detailed in Table 1. Although anesthesiologists participated in most surgical procedures, with a rate exceeding 95%, there were notable exceptions. These included anal surgeries, such as anal sphincteroplasty (23.3%), transanal rectal tumor resection (39.1%), proctocele surgery (55.9%), esophageal and gastric varix surgery (62.0%), external cholecystostomy (63.3%), external pancreatic duct drainage (73.7%), and surgery for hepatic trauma (excluding drainage only) (75.1%), which had participation rates below 80%. Although the participation rates of board‐certified surgeons vary, they generally exceed 90% in highly difficult procedures for each organ. However, even among the surgeries performed in more than 100 cases annually, procedures such as anal sphincteroplasty (53.7%), transanal rectal tumor resection (56.9%), achalasia surgery (53.4%), gastric pyloroplasty (57.5%), and surgery for hepatic trauma (excluding drainage only) (58.6%) had less than 60% participation by board‐certified surgeons.

**TABLE 1:** Characteristics of each operative procedure of the “Training Curriculum for Board‐Certified Surgeons in Gastroenterology” in 2021.

Organ	Difficulty level	Operative procedure	No. of surgeries	Sex male (%)	Age ≥80 (%)	Anesthesiologist participation (%)	Board‐certified surgeon participation (%)	Operating surgeon (%)	
								Board‐certified surgeon	Non‐board‐certified surgeon
Esophagus	Low	Cervical periesophageal abscess drainage	32	68.8	9.4	87.5	96.9	71.9	28.1
	Med	Esophageal suture (perforation, injury)	183	77.6	15.8	95.1	86.9	57.9	42.1
	Med	Thoracic periesophageal abscess drainage	23	78.3	13.0	100.0	100.0	73.9	26.1
	Med	Esophageal foreign body extraction	27	40.7	44.4	96.3	85.2	55.6	44.4
	Med	Esophageal diverticulum resection	41	63.4	9.8	95.1	95.1	78.0	22.0
	Med	Benign esophageal tumor removal	70	61.4	0.0	98.6	97.1	74.3	25.7
	Med	Esophageal resection (removal only)	606	81.2	15.3	97.4	91.4	75.6	24.4
	Med	Esophageal reconstruction (gastric tube reconstruction)	516	82.2	7.2	99.0	95.5	78.5	21.5
	Med	Esophageal fistula construction	186	77.4	12.9	96.8	96.2	83.9	16.1
	Med	Esophagocardioplasty	305	38.7	25.9	97.4	82.3	56.1	43.9
	Med	Achalasia surgery	189	52.9	7.9	96.3	53.4	38.1	61.9
	High	Esophagectomy	6193	80.3	8.4	99.2	98.6	83.5	16.5
	High	Esophageal reconstruction (colon reconstruction)	36	88.9	5.6	100.0	91.7	63.9	36.1
	High	Esophageal bypass	117	80.3	7.7	98.3	100.0	66.7	33.3
	High	Bronchoesophageal fistula surgery	7	57.1	0.0	85.7	100.0	100.0	0.0
	High	Secondary esophageal reconstruction	374	81.6	11.8	98.9	96.8	72.5	27.5
Stomach and duodenum	Low	Gastrostomy and suture gastrorrhaphy	64	60.9	21.9	96.9	82.8	40.6	59.4
	Low	Diverticulectomy, polypectomy (excluding endoscopic resection)	128	53.9	19.5	96.1	92.2	50.0	50.0
	Low	Truncal vagotomy	0	‐	‐	‐	‐	‐	‐
	Low	Gastroenterostomy (including duodenal jejunostomy)	5944	62.8	28.8	97.0	86.4	41.9	58.1
	Low	Gastric fistula construction (excluding PEG)	1460	63.9	28.0	93.1	79.9	45.5	54.5
	Low	Gastric pyloroplasty	106	79.2	3.8	96.2	57.5	28.3	71.7
	Low	Gastric volvulus surgery and rectopexy	64	20.3	48.4	95.3	84.4	46.9	53.1
	Low	Gastric suture (including gastric suture for gastric rupture, suture closure for gastroduodenal perforation, omental implantation and omental transposition)	5327	65.1	25.6	93.6	75.1	30.0	70.0
	Low	Local gastrectomy (including wedge resection)	4878	50.7	14.6	96.6	89.2	49.8	50.2
	Med	Gastrectomy (including distal, pylorus preserving, and segmental)	28 771	66.6	26.7	96.4	90.7	55.9	44.1
	Med	Selective vagotomy	3	66.7	0.0	100.0	66.7	33.3	66.7
	High	Total gastrectomy (including proximal gastrectomy)	10 010	73.8	22.6	96.5	89.4	55.6	44.4
	High	Left upper abdominal exenteration	4	100.0	0.0	100.0	100.0	75.0	25.0
Small intestine and colon	Low	Enterotomy and enterorrhaphy	4268	55.5	26.5	93.7	78.9	40.4	59.6
	Low	Disinvagination (invasive)	152	45.4	29.6	96.7	76.3	21.7	78.3
	Low	Partial small bowel resection (benign)	9017	57.5	30.9	95.0	78.9	36.6	63.4
	Low	Ileocecal resection (benign)	4970	58.6	18.4	94.6	79.0	34.4	65.6
	Low	Partial colectomy and sigmoid colectomy (benign)	8536	62.2	25.9	95.0	80.8	37.3	62.7
	Low	Appendectomy	56 071	55.1	6.6	93.1	68.3	24.3	75.7
	Low	Enterostomy and closure (without enterectomy)	28 205	62.8	20.4	96.0	83.3	45.0	55.0
	Med	Small bowel resection (malignant)	3620	56.8	20.4	97.1	85.2	42.1	57.9
	Med	Ileocecal resection (malignant)	15 488	46.2	33.6	96.1	86.0	37.3	62.7
	Med	Partial colectomy and sigmoid colectomy (malignant)	30 536	57.2	24.9	96.3	87.1	46.8	53.2
	Med	Right hemicolectomy	22 027	51.0	33.9	95.8	85.3	44.1	55.9
	Med	Left hemicolectomy	6018	57.6	24.9	95.9	85.9	49.2	50.8
	Med	Total colectomy	1546	58.2	21.8	96.2	84.0	49.9	50.1
	Med	Intestinal obstruction surgery (with enterectomy)	26 275	49.2	37.9	94.4	76.6	33.8	66.2
	Med	Enterostomy and closure (with enterectomy)	23 231	62.9	18.5	95.9	82.7	42.2	57.8
	High	Proctocolectomy and ileoanal (canal) anastomosis	440	61.8	1.4	99.3	95.7	77.0	23.0
Rectum	Low	Transanal rectal tumor resection	3527	49.6	12.6	39.1	56.9	36.1	63.9
	Low	Proctocele surgery (transanal)	2396	14.2	66.0	55.9	63.1	37.9	62.1
	Med	Abdominoperineal resection (benign)	1056	59.8	21.2	93.7	82.1	48.3	51.7
	Med	High anterior resection	11 841	58.1	18.3	96.7	89.1	54.3	45.7
	Med	Hartmann's procedure	6408	55.9	38.5	96.3	83.1	39.4	60.6
	Med	Proctocele surgery (abdominoperineal)	2198	10.7	58.9	91.9	83.9	51.5	48.5
	Med	Malignant anorectal tumor excision (transanal)	706	52.5	30.3	80.0	79.7	56.1	43.9
	Med	Anal sphincteroplasty (by tissue replacement)	2603	54.9	11.6	23.3	53.7	37.2	62.8
	High	Abdominoperineal resection (malignant)	4909	63.5	21.3	96.7	91.2	66.1	33.9
	High	Low anterior resection	20 403	62.8	14.1	96.3	90.7	65.9	34.1
	High	Total pelvic exenteration	454	71.8	4.8	98.5	92.7	73.6	26.4
	High	Anorectal malignant tumor excision (posterior approach)	35	54.3	20.0	80.0	88.6	48.6	51.4
Liver	Low	Hepatorrhaphy	63	55.6	27.0	87.3	69.8	27.0	73.0
	Low	Liver abscess drainage (excluding percutaneous procedures)	40	67.5	27.5	85.0	95.0	45.0	55.0
	Low	Hepatic cyst resection, suture, drainage	966	24.0	18.5	97.0	88.5	43.7	56.3
	Low	Liver biopsy (excluding percutaneous procedures)	351	49.0	2.6	91.2	88.0	39.0	61.0
	Low	Liver coagulo‐necrotic therapy (excluding percutaneous procedures)	439	72.9	19.1	98.4	92.9	66.3	33.7
	Med	Partial hepatectomy	12 492	66.7	15.7	98.0	95.7	69.5	30.5
	Med	Lateral segmentectomy	1382	65.9	20.3	97.7	94.9	64.5	35.5
	Med	Esophageal and gastric varix surgery	50	62.0	10.0	62.0	40.0	32.0	68.0
	High	Surgery for hepatic trauma (excluding drainage only)	394	67.3	19.3	75.1	58.6	27.4	72.6
	High	Hepatectomy (segmentectomy or more; excluding lateral segmentectomy)	6491	67.8	15.4	98.2	97.6	80.2	19.8
	High	Subsegmentectomy	2656	70.8	16.8	97.3	97.0	79.0	21.0
	High	Liver transplantation	791	51.2	0.3	99.1	98.9	77.2	22.8
	High	Hepatopancreatoduodenectomy	135	67.4	5.2	96.3	99.3	88.9	11.1
Gall bladder	Low	Cholangiotomy	63	57.1	33.3	98.4	90.5	47.6	52.4
	Low	Cholecystolithotomy	64	62.5	37.5	92.2	84.4	45.3	54.7
	Low	Cholecystectomy	129 693	55.0	17.7	95.0	80.0	34.9	65.1
	Low	External cholecystostomy	139	60.4	47.5	63.3	65.5	36.0	64.0
	Low	Cystoenteric anastomosis	36	52.8	52.8	97.2	86.1	55.6	44.4
	Med	Choledocholithotomy	2015	60.4	38.5	94.1	85.5	48.2	51.8
	Med	Biliary tract reconstruction	295	57.6	20.3	96.9	96.3	70.2	29.8
	Med	Biliary bypass	967	125.9	26.8	97.2	94.1	57.5	42.5
	Med	Cholangioplasty	96	#REF!	21.9	92.7	91.7	63.5	36.5
	Med	Duodenal papilloplasty	29	58.6	27.6	82.8	93.1	72.4	27.6
	Med	Choledocal dilatation	278	25.9	2.2	97.8	91.0	66.2	33.8
	Med	Biliary fistula closure	16	50.0	43.8	93.8	87.5	31.3	68.8
	High	Surgery for bile duct trauma (excluding drainage only)	235	58.7	25.5	95.7	90.2	53.6	46.4
	High	Malignant gallbladder tumor surgery (excluding simple cholecystectomy)	1039	51.9	23.1	98.5	94.9	61.2	38.8
	High	Malignant bile duct tumor surgery	1108	69.4	23.1	96.9	97.3	81.3	18.7
	High	Biliary atresia surgery	38	39.5	0.0	100.0	50.0	21.1	78.9
Pancreas	Low	External pancreatic cyst drainage	4	100.0	0.0	100.0	25.0	25.0	75.0
	Low	External pancreatic duct drainage	19	73.7	10.5	73.7	78.9	73.7	26.3
	Med	Pancreatorrhaphy	4	100.0	25.0	100.0	75.0	75.0	25.0
	Med	Partial pancreatic resection	145	46.9	5.5	98.6	95.2	77.2	22.8
	Med	Distal pancreatectomy (benign)	1422	46.8	9.1	97.3	94.4	74.0	26.0
	Med	Pancreatic cyst‐enterostomy	20	70.0	15.0	95.0	85.0	55.0	45.0
	Med	Pancreatic (duct) enterostomy	280	70.4	8.2	94.6	97.5	69.3	30.7
	Med	Acute pancreatitis surgery	50	64.0	8.0	94.0	80.0	54.0	46.0
	Med	Pancreatolithiasis surgery	14	78.6	7.1	100.0	85.7	71.4	28.6
	Med	Plexus pancreaticus capitalis resection	0	‐	‐	‐	‐	‐	‐
	High	Surgery for pancreatic trauma (excluding drainage only)	59	69.5	13.6	93.2	74.6	50.8	49.2
	High	Pancreaticoduodenectomy	11 764	60.2	16.7	98.1	97.4	75.5	24.5
	High	Distal pancreatectomy (malignant)	5091	55.6	17.8	97.8	96.5	75.3	24.7
	High	Total pancreatectomy	635	54.3	12.1	98.7	98.7	81.3	18.7
	High	Duodenum preserving pancreas head resection	38	47.4	5.3	97.4	89.5	60.5	39.5
	High	Segmental pancreatic resection	153	50.3	5.2	98.7	99.3	76.5	23.5
	High	Pancreas transplantation	23	60.9	0.0	100.0	100.0	95.7	4.3
Spleen	Low	Splenorrhaphy	24	62.5	16.7	91.7	66.7	25.0	75.0
	Med	Splenectomy	1789	55.9	12.7	96.4	91.2	61.3	38.7
	Med	Partial splenectomy	20	45.0	10.0	95.0	45.0	20.0	80.0
Other	Low	Localized intra‐abdominal abscess surgery	2243	60.5	17.9	92.0	75.8	34.5	65.5
	Low	Exploratory laparotomy	13 325	60.6	18.6	94.8	82.5	42.8	57.2
	Med	Acute diffuse peritonitis surgery	15 776	58.8	28.0	95.1	80.3	34.4	65.6
	Med	Ventral hernia surgery	14 399	46.5	21.1	94.4	74.4	37.4	62.6
	Med	Diaphragm suture	285	56.5	22.1	96.8	83.2	51.6	48.4
	Med	Esophageal hiatus hernia surgery	1231	31.0	41.0	96.2	89.7	61.7	38.3
	Med	Retroperitoneal tumor surgery	1425	49.0	9.9	97.4	88.8	60.3	39.7
	Med	Abdominal wall/mesenteric/omental tumor resection	2085	50.3	10.2	96.5	82.4	46.8	53.2
	Med	Gastrointestinal perforation closure	391	65.7	26.3	92.8	80.3	38.1	61.9
	High	Diaphragmatic hiatus hernia surgery	56	57.1	23.2	96.4	92.9	55.4	44.6

The short‐term outcomes of the operative procedures outlined in the “Training Curriculum for Board‐Certified Surgeons in Gastroenterology” in 2021 are presented in Table 2. Among the procedures performed in more than 100 cases annually, those with high surgical mortality rates (>10%) included surgery for hepatic trauma (excluding drainage only) (26.6%), closure of gastrointestinal perforations (16.2%), surgery for acute diffuse peritonitis (11.5%), esophageal bypass (11.1%), esophageal fistula construction (10.8%), and external cholecystostomy (10.1%). These procedures predominantly involved emergency surgeries, and a high proportion of patients experienced palliative or symptomatic relief.

**TABLE 2:** Number of surgeries and short‐term outcome of each operative procedure of the “Training Curriculum for Board‐Certified Surgeons in Gastroenterology” in 2021.

Organ	Difficulty level	Operative procedure	No. of surgeries	Endoscopic surgeries (%)	Emergency surgeries (%)	Postoperative complications (%)^a^	Re‐operations (%)	Postoperative 30‐day mortalities (%)	Operative mortalities (%)^b^
Esophagus	Low	Cervical periesophageal abscess drainage	32	21.9	59.4	28.1	9.4	3.1	3.1
	Med	Esophageal suture (perforation, injury)	183	19.1	74.9	36.6	13.7	3.3	4.9
	Med	Thoracic periesophageal abscess drainage	23	17.4	91.3	21.7	30.4	4.3	8.7
	Med	Esophageal foreign body extraction	27	22.2	88.9	22.2	0.0	0.0	3.7
	Med	Esophageal diverticulum resection	41	34.1	0.0	12.2	4.9	0.0	0.0
	Med	Benign esophageal tumor removal	70	81.4	0.0	0.0	2.9	0.0	0.0
	Med	Esophageal resection (removal only)	606	63.5	8.7	20.1	16.5	1.5	3.8
	Med	Esophageal reconstruction (gastric tube reconstruction)	516	71.7	1.2	21.5	5.0	1.0	3.1
	Med	Esophageal fistula construction	186	49.5	26.9	39.8	31.2	2.2	10.8
	Med	Esophagocardioplasty	305	76.7	2.6	4.6	2.0	1.0	1.0
	Med	Achalasia surgery	189	91.5	0.0	0.0	0.0	0.0	0.0
	High	Esophagectomy	6193	74.8	0.9	23.0	5.7	0.7	1.4
	High	Esophageal reconstruction (colon reconstruction)	36	55.6	0.0	25.0	16.7	2.8	2.8
	High	Esophageal bypass	117	12.0	2.6	32.5	9.4	6.0	11.1
	High	Bronchoesophageal fistula surgery	7	14.3	14.3	57.1	14.3	0.0	0.0
	High	Secondary esophageal reconstruction	374	15.2	2.1	32.9	10.4	0.8	2.4
Stomach and duodenum	Low	Gastrostomy and suture gastrorrhaphy	64	14.1	62.5	10.9	3.1	0.0	0.0
	Low	Diverticulectomy, polypectomy (excluding endoscopic resection)	128	16.4	23.4	13.3	5.5	1.6	2.3
	Low	Truncal vagotomy	0	‐	‐	‐	‐	‐	‐
	Low	Gastroenterostomy (including duodenal jejunostomy)	5944	33.6	9.7	15.1	5.2	5.0	8.5
	Low	Gastric fistula construction (excluding PEG)	1460	15.4	16.5	20.5	4.7	6.3	9.8
	Low	Gastric pyloroplasty	106	17.0	43.4	4.7	5.7	0.9	0.9
	Low	Gastric volvulus surgery and rectopexy	64	71.9	20.3	14.1	4.7	1.6	1.6
	Low	Gastric suture (including gastric suture for gastric rupture, suture closure for gastroduodenal perforation, omental implantation and omental transposition)	5327	38.6	91.5	17.7	5.7	5.7	7.5
	Low	Local gastrectomy (including wedge resection)	4878	71.3	3.1	3.1	1.4	0.3	0.5
	Med	Gastrectomy (including distal, pylorus preserving and segmental)	28 771	56.3	1.8	7.5	2.5	0.7	1.1
	Med	Selective vagotomy	3	66.7	0.0	0.0	0.0	0.0	0.0
	High	Total gastrectomy (including proximal gastrectomy)	10 010	33.8	1.9	12.5	3.9	1.3	2.2
	High	Left upper abdominal exenteration	4	0.0	0.0	75.0	25.0	0.0	0.0
Small intestine and colon	Low	Enterotomy and enterorrhaphy	4268	18.6	29.7	17.0	7.7	4.5	7.5
	Low	Disinvagination (invasive)	152	29.6	80.3	5.3	2.0	2.6	3.3
	Low	Partial small bowel resection (benign)	9017	21.1	62.5	20.4	10.4	6.7	9.1
	Low	Ileocecal resection (benign)	4970	44.7	49.1	10.3	4.1	2.2	3.0
	Low	Partial colectomy and sigmoid colectomy (benign)	8536	35.6	45.3	15.5	6.3	4.1	5.6
	Low	Appendectomy	56 071	73.9	67.6	1.8	1.0	0.1	0.2
	Low	Enterostomy and closure (without enterectomy)	28 205	40.7	28.4	16.0	7.6	3.5	5.7
	Med	Small bowel resection (malignant)	3620	32.2	17.4	12.1	4.8	2.8	4.2
	Med	Ileocecal resection (malignant)	15 488	65.2	5.7	4.9	2.2	0.6	0.9
	Med	Partial colectomy and sigmoid colectomy (malignant)	30 536	64.8	3.6	6.3	3.6	0.5	0.9
	Med	Right hemicolectomy	22 027	56.9	8.2	7.4	3.4	1.4	2.1
	Med	Left hemicolectomy	6018	56.1	9.2	10.1	4.8	2.0	2.9
	Med	Total colectomy	1546	33.1	33.4	23.9	9.5	7.9	9.8
	Med	Intestinal obstruction surgery (with enterectomy)	26 275	25.7	68.3	10.2	4.4	2.4	3.4
	Med	Enterostomy and closure (with enterectomy)	23 231	19.4	21.2	13.6	5.3	3.2	4.5
	High	Proctocolectomy and ileoanal (canal) anastomosis	440	52.7	7.7	10.5	4.3	0.9	1.1
Rectum	Low	Transanal rectal tumor resection	3527	2.4	1.4	0.7	0.8	0.1	0.1
	Low	Proctocele surgery (transanal)	2396	0.8	1.3	1.9	2.5	0.2	0.2
	Med	Abdominoperineal resection (benign)	1056	13.1	19.3	18.4	6.6	2.5	3.8
	Med	High anterior resection	11 841	74.9	3.4	5.6	3.5	0.4	0.6
	Med	Hartmann's procedure	6408	23.3	58.6	20.8	6.0	5.6	7.5
	Med	Proctocele surgery (abdominoperineal)	2198	58.6	0.7	2.2	1.6	0.2	0.4
	Med	Malignant anorectal tumor excision (transanal)	706	19.5	7.4	7.2	3.8	1.0	1.4
	Med	Anal sphincteroplasty (by tissue replacement)	2603	1.8	2.2	0.8	1.2	0.0	0.1
	High	Abdominoperineal resection (malignant)	4909	73.9	0.8	12.3	4.7	0.6	0.8
	High	Low anterior resection	20 403	75.3	1.3	10.4	6.1	0.5	0.6
	High	Total pelvic exenteration	454	33.0	1.5	32.4	8.8	0.9	1.8
	High	Anorectal malignant tumor excision (posterior approach)	35	14.3	0.0	11.4	5.7	0.0	0.0
Liver	Low	Hepatorrhaphy	63	11.1	85.7	34.9	23.8	11.1	12.7
	Low	Liver abscess drainage (excluding percutaneous procedures)	40	32.5	42.5	20.0	2.5	5.0	5.0
	Low	Hepatic cyst resection, suture, drainage	966	79.3	5.5	2.8	1.2	0.2	0.2
	Low	Liver biopsy (excluding percutaneous procedures)	351	13.7	26.2	4.0	7.4	0.6	1.4
	Low	Liver coagulo‐necrotic therapy (excluding percutaneous procedures)	439	33.5	0.5	3.4	2.1	0.2	0.9
	Med	Partial hepatectomy	12 492	43.9	0.7	6.4	1.9	0.4	0.5
	Med	Lateral segmentectomy	1382	41.6	1.5	5.2	2.1	0.4	0.7
	Med	Esophageal and gastric varix surgery	50	48.0	20.0	6.0	14.0	2.0	2.0
	High	Surgery for hepatic trauma (excluding drainage only)	394	5.3	82.0	48.5	49.0	22.8	26.6
	High	Hepatectomy (segmentectomy or more; excluding lateral segmentectomy)	6491	17.7	0.5	15.1	2.5	1.3	2.1
	High	Subsegmentectomy	2656	34.4	0.3	7.4	1.3	0.3	0.4
	High	Liver transplantation	791	0.6	10.1	23.1	12.6	2.1	3.5
	High	Hepatopancreatoduodenectomy	135	0.0	0.7	51.1	8.9	4.4	8.9
Gall bladder	Low	Cholangiotomy	63	11.1	22.2	15.9	3.2	1.6	1.6
	Low	Cholecystolithotomy	64	28.1	15.6	10.9	4.7	0.0	0.0
	Low	Cholecystectomy	129 693	72.9	16.3	3.6	1.1	0.4	0.6
	Low	External cholecystostomy	139	28.8	51.8	24.5	14.4	4.3	10.1
	Low	Cystoenteric anastomosis	36	11.1	5.6	22.2	5.6	5.6	11.1
	Med	Choledocholithotomy	2015	34.9	14.0	9.1	0.1	1.2	1.9
	Med	Biliary tract reconstruction	295	5.8	3.1	20.7	4.7	1.7	3.1
	Med	Biliary bypass	967	4.9	10.9	16.9	5.2	2.0	3.4
	Med	Cholangioplasty	96	12.5	18.8	25.0	2.1	2.1	2.1
	Med	Duodenal papilloplasty	29	13.8	3.4	10.3	10.3	3.4	3.4
	Med	Choledocal dilatation	278	26.3	1.8	9.0	3.2	0.4	0.4
	Med	Biliary fistula closure	16	31.3	43.8	31.3	12.5	6.3	18.8
	High	Surgery for bile duct trauma (excluding drainage only)	235	22.1	52.8	27.2	11.1	5.1	6.8
	High	Malignant gallbladder tumor surgery (excluding simple cholecystectomy)	1039	7.6	0.7	11.8	1.8	0.3	0.5
	High	Malignant bile duct tumor surgery	1108	0.8	0.5	33.6	5.6	3.2	5.7
	High	Biliary atresia surgery	38	47.4	28.9	21.1	10.5	0.0	0.0
Pancreas	Low	External pancreatic cyst drainage	4	0.0	25.0	0.0	25.0	0.0	0.0
	Low	External pancreatic duct drainage	19	0.0	63.2	57.9	31.6	5.3	21.1
	Med	Pancreatorrhaphy	4	0.0	100.0	75.0	25.0	0.0	25.0
	Med	Partial pancreatic resection	145	35.9	0.7	16.6	2.1	0.0	0.7
	Med	Distal pancreatectomy (benign)	1422	52.3	4.1	16.8	2.3	0.7	0.9
	Med	Pancreatic cyst‐enterostomy	20	5.0	10.0	25.0	5.0	10.0	10.0
	Med	Pancreatic (duct) enterostomy	280	0.0	7.5	18.6	4.6	2.1	3.6
	Med	Acute pancreatitis surgery	50	16.0	58.0	52.0	14.0	6.0	16.0
	Med	Pancreatolithiasis surgery	14	0.0	0.0	0.0	0.0	0.0	0.0
	Med	Plexus pancreaticus capitalis resection	0	‐	‐	‐	‐	‐	‐
	High	Surgery for pancreatic trauma (excluding drainage only)	59	5.1	78.0	44.1	25.4	8.5	13.6
	High	Pancreaticoduodenectomy	11 764	5.2	0.7	24.1	3.3	0.9	1.6
	High	Distal pancreatectomy (malignant)	5091	33.4	0.8	20.0	2.1	0.3	0.7
	High	Total pancreatectomy	635	1.9	3.5	15.7	4.6	1.9	3.0
	High	Duodenum preserving pancreas head resection	38	0.0	0.0	26.3	2.6	2.6	5.3
	High	Segmental pancreatic resection	153	8.5	1.3	34.0	2.0	0.7	0.7
	High	Pancreas transplantation	23	0.0	73.9	34.8	43.5	0.0	4.3
Spleen	Low	Splenorrhaphy	24	16.7	87.5	29.2	29.2	8.3	8.3
	Med	Splenectomy	1789	29.9	1.2	14.6	5.5	2.6	3.7
	Med	Partial splenectomy	20	35.0	20.0	10.0	15.0	0.0	0.0
Other	Low	Localized intra‐abdominal abscess surgery	2376	32.7	70.5	14.6	6.6	2.2	3.2
	Low	Exploratory laparotomy	12 401	49.3	30.3	16.8	13.2	6.2	9.0
	Med	Acute diffuse peritonitis surgery	15 542	22.1	92.6	27.3	7.8	8.0	11.5
	Med	Ventral hernia surgery	14 136	32.0	11.4	3.9	2.0	0.6	0.9
	Med	Diaphragm suture	294	34.0	40.5	19.7	9.5	4.4	6.5
	Med	Esophageal hiatus hernia surgery	1217	62.8	7.9	8.0	4.1	1.4	2.2
	Med	Retroperitoneal tumor surgery	1551	10.9	2.1	9.2	3.5	0.3	0.5
	Med	Abdominal wall/mesenteric/omental tumor resection	2054	31.3	15.3	7.7	4.3	0.9	1.9
	Med	Gastrointestinal perforation closure	421	11.9	90.5	35.4	14.0	11.6	16.2
	High	Diaphragmatic hiatus hernia surgery	56	37.5	42.9	12.5	3.6	10.7	12.5

^a^ Complications were defined by Clavien–Dindo grade IIIa‐V.

^b^ Operative mortality was a rate that combined 30‐day mortality and hospitalization death in 31–90 days after surgery.

### 3.2 Annual trends in organ‐specific surgical procedures

**FIGURE 1:**
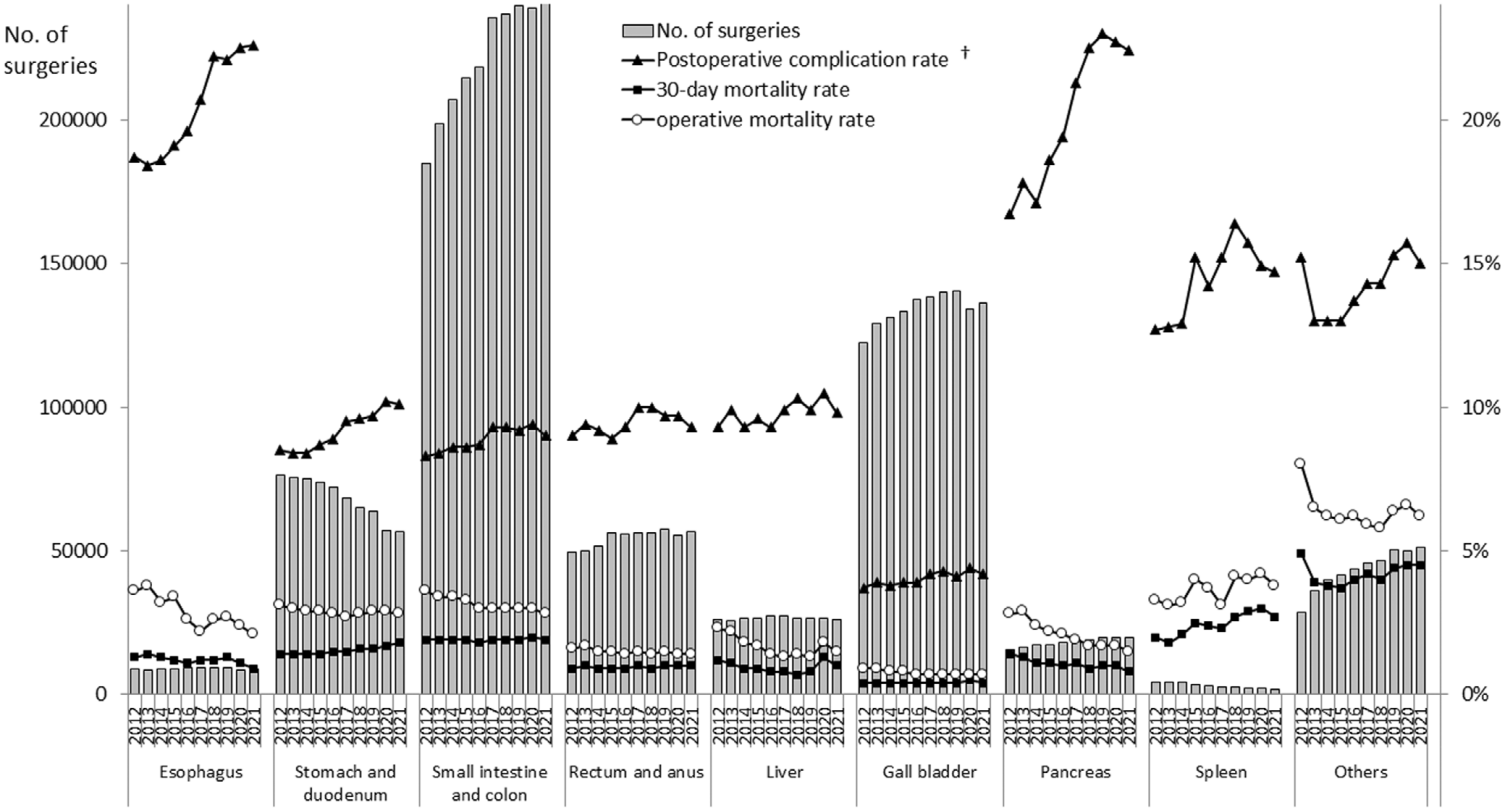
Annual changes in the number of surgeries, 30‐day and operative mortality rates, and complication rates of each organ. †Postoperative complication was defined as grades IIIa–V in the Clavien–Dindo classification.

Figure 1 illustrates the trends in the number of gastroenterological surgeries and key outcomes in Japan from 2012 to 2021. Long‐term trends indicate an increasing trend in the number of surgeries for five regions: the small intestine and colon, rectum and anus, gallbladder, pancreas, and the “others” classification. Conversely, the number of surgeries for the esophagus and liver remained stable, while those for the stomach and duodenum, and spleen showed decreasing trends. Notably, from a short‐term perspective, there was a decrease in the number of surgeries across all areas in 2020; however, the numbers rebounded, except for that of surgeries for the stomach and duodenum, liver, and spleen.

Table 3 summarizes the sex ratio and age distribution by organ in surgeries performed over a decade. The proportion of female patients increased in all organs, with the most significant increases observed in the spleen, esophagus, stomach, and duodenum. Across all organs, there has been an increase in the proportion of patients aged ≥70 years, particularly those >80 years. Conversely, the proportion of patients <60 years of age generally decreased, except for surgeries for the rectum and anus, wherein the proportion slightly increased.

**TABLE 3:** Annual changes in surgeries of each organ by sex and age of patients.

Organ	Year	No. of surgeries	Sex (%)		Age (%)					
			Male	Female	<60	60 to 64	65–69	70–74	75–79	≥80
Esophagus	2012	8819	82.2	17.8	22.1	19.7	20.0	19.5	12.9	6.0
	2013	8642	81.5	18.5	20.8	17.5	21.0	20.6	13.2	6.9
	2014	9021	81.5	18.4	20.8	16.5	21.4	20.9	13.8	6.6
	2015	8943	80.8	19.2	19.6	15.3	22.4	22.5	13.1	7.1
	2016	9212	79.6	20.4	20.1	14.4	22.9	20.5	14.5	7.5
	2017	9359	80.0	20.0	19.3	13.4	24.4	19.4	15.5	8.0
	2018	9286	78.4	21.6	19.0	12.8	21.3	21.6	16.7	8.7
	2019	9224	78.6	21.4	18.8	13.1	19.4	22.8	17.3	8.6
	2020	8713	79.0	21.0	18.4	13.5	18.3	23.5	16.5	9.8
	2021	8905	78.0	22.0	19.0	12.5	17.8	24.4	16.5	9.8
Stomach and duodenum	2012	76 186	68.3	31.7	18.9	14.4	14.5	17.1	16.4	18.6
	2013	75 583	67.9	32.1	18.6	13.1	15.5	17.2	16.9	18.7
	2014	74 920	67.6	32.4	17.9	12.1	16.0	17.8	16.7	19.5
	2015	73 877	67.8	32.2	17.4	11.1	17.1	17.8	16.6	19.9
	2016	72 234	67.8	32.2	17.0	10.2	18.1	17.1	16.6	21.0
	2017	68 287	67.2	32.8	16.3	9.9	17.5	17.3	17.2	21.8
	2018	65 152	66.9	33.1	16.0	9.0	16.4	18.0	17.5	23.2
	2019	63 610	66.5	33.5	15.6	8.8	15.0	19.0	18.5	23.2
	2020	57 171	66.6	33.4	15.3	8.2	13.8	20.1	18.5	24.1
	2021	56 759	65.9	34.1	15.7	7.7	12.6	20.9	18.1	25.0
Small intestine and colon	2012	184 810	56.7	43.3	36.4	10.7	10.7	12.2	12.5	17.4
	2013	198 677	56.9	43.1	35.6	10.1	11.3	12.7	12.4	17.8
	2014	206 857	56.9	43.1	34.7	9.4	12.0	13.1	12.4	18.4
	2015	214 453	57.1	42.9	34.0	8.9	12.9	13.1	12.3	18.7
	2016	218 228	57.3	42.7	33.7	8.4	13.6	12.5	12.4	19.3
	2017	235 359	56.7	43.3	32.7	8.0	13.2	12.7	12.9	20.5
	2018	236 496	56.9	43.1	32.2	7.7	12.6	13.4	13.2	21.1
	2019	239 612	56.3	43.7	32.1	7.4	11.7	13.9	13.5	21.2
	2020	238 631	56.2	43.8	32.6	7.3	10.7	14.6	13.4	21.5
	2021	240 448	56.0	44.0	31.9	7.3	10.3	15.5	12.7	22.3
Rectum and anus	2012	49 704	58.3	41.7	22.3	14.8	14.6	15.5	14.3	18.5
	2013	49 980	58.0	42.0	20.9	13.9	15.2	16.1	14.6	19.3
	2014	51 454	58.3	41.7	20.4	13.1	16.0	16.4	14.2	19.9
	2015	56 092	57.8	42.2	22.3	11.8	16.7	15.7	14.0	19.4
	2016	55 666	57.3	42.7	22.0	11.1	17.9	15.0	13.6	20.4
	2017	56 144	56.7	43.3	22.2	10.2	17.3	15.1	14.2	21.0
	2018	56 162	56.9	43.1	22.2	9.8	15.9	15.8	14.6	21.6
	2019	57 706	56.3	43.7	22.5	9.5	14.8	16.5	14.9	21.9
	2020	55 536	56.2	43.8	22.7	9.2	13.7	17.6	14.7	22.1
	2021	56 536	55.7	44.3	23.1	9.0	12.8	18.7	14.1	22.3
Liver	2012	26 288	66.3	33.7	22.1	15.7	16.7	18.0	17.4	10.2
	2013	25 814	66.1	33.9	21.3	14.6	17.6	18.7	17.3	10.5
	2014	26 518	66.3	33.7	21.5	13.7	18.1	19.8	16.6	10.3
	2015	26 378	65.7	34.3	20.8	12.8	18.9	19.4	16.5	11.5
	2016	27 212	66.4	33.6	20.3	11.5	20.5	18.6	17.0	12.1
	2017	27 397	65.8	34.2	20.1	11.0	20.2	18.8	17.2	12.7
	2018	26 531	66.5	33.5	19.6	10.3	18.8	19.6	17.8	13.8
	2019	26 582	66.3	33.7	19.4	10.1	16.5	21.1	18.6	14.2
	2020	26 614	66.0	34.0	20.6	9.5	15.1	21.7	18.7	14.4
	2021	26 250	65.2	34.8	20.8	9.9	14.1	22.4	17.3	15.5
Gall bladder	2012	122 513	55.2	44.8	32.9	13.8	12.4	13.9	13.2	13.8
	2013	129 162	55.3	44.7	32.6	12.9	13.0	14.2	13.2	14.0
	2014	131 182	55.6	44.4	32.1	11.8	13.9	14.5	13.2	14.5
	2015	133 126	55.6	44.4	32.0	11.2	15.0	14.1	13.0	14.8
	2016	137 360	55.4	44.6	32.6	10.6	15.5	13.1	12.9	15.3
	2017	138 267	55.6	44.4	32.2	10.2	15.1	13.5	13.2	15.8
	2018	139 844	55.3	44.7	31.8	9.7	14.2	14.2	13.4	16.7
	2019	140 214	55.4	44.6	31.6	9.6	13.3	14.7	13.9	16.9
	2020	134 332	55.9	44.1	31.3	9.1	12.2	15.6	14.0	17.8
	2021	136 111	55.2	44.8	31.7	9.1	11.5	16.6	13.0	18.2
Pancreas	2012	15 550	60.0	40.0	19.8	15.2	17.0	19.5	18.2	10.3
	2013	16 380	59.7	40.3	19.1	13.6	18.0	20.7	17.7	10.9
	2014	17 313	59.5	40.5	18.4	12.4	19.0	21.0	18.2	11.1
	2015	17 407	59.1	40.9	18.2	11.3	19.4	21.6	18.1	11.4
	2016	18 238	58.9	41.1	18.2	10.4	19.9	20.4	19.0	12.2
	2017	19 138	59.2	40.8	17.7	9.9	19.5	19.9	20.1	12.9
	2018	19 152	58.6	41.4	16.9	9.2	18.2	21.5	20.4	13.7
	2019	19 703	58.3	41.7	17.0	9.2	16.5	21.6	21.1	14.6
	2020	19 947	58.1	41.9	16.7	8.4	14.6	22.8	21.9	15.6
	2021	19 722	57.9	42.1	17.0	8.4	13.7	24.2	20.8	15.9
Spleen	2012	4142	61.4	38.6	32.9	16.3	15.0	15.1	12.9	7.8
	2013	4509	61.8	38.2	30.8	14.9	15.9	16.5	13.1	8.7
	2014	4272	61.8	38.2	29.9	13.0	17.3	17.0	13.8	9.1
	2015	3568	60.4	39.6	29.7	11.4	17.3	16.6	14.1	10.8
	2016	3171	57.3	42.7	31.9	11.7	17.7	15.7	12.5	10.5
	2017	2864	58.7	41.3	31.6	11.0	18.1	16.0	13.3	10.0
	2018	2544	56.6	43.4	32.6	9.9	15.6	16.9	13.9	11.1
	2019	2413	55.2	44.8	31.3	10.5	16.8	15.8	13.1	12.5
	2020	2096	54.4	45.6	32.8	11.4	12.6	16.7	14.1	12.4
	2021	1833	55.9	44.1	32.0	9.8	13.4	17.5	14.7	12.7
Others	2012	28 779	55.4	44.6	31.1	11.7	11.7	13.8	13.7	18.0
	2013	36 363	53.1	46.9	28.3	10.9	12.7	14.1	14.8	19.1
	2014	39 854	53.7	46.3	28.1	10.1	13.1	14.5	14.4	19.8
	2015	41 465	53.2	46.8	27.4	9.4	14.0	14.5	14.2	20.6
	2016	43 523	54.0	46.0	27.5	9.2	14.6	13.5	14.0	21.2
	2017	45 622	54.1	45.9	27.0	8.2	14.7	13.5	14.6	21.9
	2018	46 587	54.1	45.9	26.8	8.2	14.0	14.4	14.7	21.9
	2019	50 525	54.8	45.2	27.0	8.1	12.7	15.3	15.0	21.9
	2020	50 048	54.5	45.5	27.2	7.9	11.9	16.0	14.9	22.1
	2021	51 216	54.6	45.4	27.6	7.8	11.3	17.1	14.0	22.2

Table 4 compiles the data on the institutions where surgeries were performed and the participation rates of anesthesiologists and board‐certified surgeons. Long‐term trends indicate that surgeries for all organs are increasingly being performed at certified or affiliated institutions. However, in 2021, there was a slight decrease in the number of spleen and liver surgeries performed at these institutions. The participation rates of anesthesiologists and board‐certified surgeons in surgery increased annually across all areas. Notably, the participation rate of anesthesiologists in rectal and anal surgeries was approximately 87%, which was approximately 10% lower than that in other areas. Furthermore, the involvement of board‐certified surgeons in surgeries of the esophagus, liver, and pancreas is exceptionally high, exceeding 95%.

**TABLE 4:** Annual changes in surgeries of each organ by institution type and specialist participation rate.

Organ	Year	No. of surgeries	Institution type (%)			Anesthesiologist participation (%)	Board‐certified surgeon participation (%)	Operating surgeon (%)	
			Certified institution	Affiliated institution	Others			Board‐certified surgeon	Non‐board‐ certified surgeon
Esophagus	2012	8819	78.1	5.9	16.0	97.4	87.0	62.7	37.3
	2013	8642	90.6	7.1	2.4	97.3	88.4	64.4	35.6
	2014	9021	91.1	6.1	2.8	97.9	90.1	67.6	32.4
	2015	8943	91.5	6.0	2.5	97.9	91.1	69.4	30.6
	2016	9212	92.4	5.0	2.6	98.2	91.2	70.0	30.0
	2017	9359	92.7	4.0	3.3	97.9	92.5	71.8	28.2
	2018	9286	93.8	4.0	2.2	98.5	94.7	75.2	24.8
	2019	9224	94.3	3.8	1.9	98.4	94.2	76.4	23.6
	2020	8713	95.2	3.2	1.5	98.9	95.7	78.3	21.7
	2021	8905	95.9	2.7	1.4	98.7	96.0	79.2	20.8
Stomach and duodenum	2012	76 186	63.5	15.6	20.9	93.5	70.3	35.6	64.4
	2013	75 583	76.3	19.3	4.4	93.3	73.5	37.7	62.3
	2014	74 920	77.0	18.2	4.8	93.6	75.9	39.2	60.8
	2015	73 877	77.1	18.3	4.6	93.9	76.1	39.2	60.8
	2016	72 234	79.6	16.1	4.3	94.6	78.7	41.0	59.0
	2017	68 287	79.6	15.3	5.1	94.8	79.7	41.8	58.2
	2018	65 152	80.0	14.8	5.1	95.1	81.4	43.2	56.8
	2019	63 610	81.3	14.2	4.5	95.4	83.8	46.1	53.9
	2020	57 171	80.8	14.8	4.4	95.7	85.4	47.6	52.4
	2021	56 759	81.3	14.2	4.4	96.2	88.1	51.1	48.9
Small intestine and colon	2012	184 810	60.6	18.2	21.2	88.9	59.9	25.4	74.6
	2013	198 677	72.6	22.2	5.2	89.6	62.7	26.6	73.4
	2014	206 857	73.0	21.4	5.6	90.8	65.4	28.1	71.9
	2015	214 453	73.8	20.7	5.5	91.6	66.3	28.5	71.5
	2016	218 228	75.6	19.0	5.5	92.4	68.1	29.5	70.5
	2017	235 359	76.0	18.0	6.0	92.9	70.1	31.1	68.9
	2018	236 496	76.3	17.5	6.1	93.3	71.8	32.6	67.4
	2019	239 612	77.1	17.1	5.8	94.1	74.0	33.2	66.8
	2020	238 631	76.5	17.9	5.6	94.5	75.9	34.2	65.8
	2021	240 448	77.5	16.9	5.6	95.1	79.5	37.6	62.4
Rectum and anus	2012	49 704	60.4	18.2	21.4	85.7	68.6	37.6	62.4
	2013	49 980	72.9	21.7	5.4	87.3	71.2	39.4	60.6
	2014	51 454	73.5	20.9	5.6	87.9	73.7	41.6	58.4
	2015	56 092	72.5	20.8	6.7	84.9	73.5	41.5	58.5
	2016	55 666	74.1	19.4	6.6	85.7	74.7	42.1	57.9
	2017	56 144	73.8	18.2	8.0	84.8	76.1	43.9	56.1
	2018	56 162	74.1	17.9	8.0	85.2	77.2	46.7	53.3
	2019	57 706	74.9	17.3	7.8	86.0	80.1	48.9	51.1
	2020	55 536	74.5	18.6	6.8	86.7	81.7	51.0	49.0
	2021	56 536	75.8	17.6	6.6	87.4	84.0	55.2	44.8
Liver	2012	26 288	74.2	9.2	16.7	95.4	85.7	57.4	42.6
	2013	25 814	86.3	10.7	2.9	96.3	87.5	57.1	42.9
	2014	26 518	86.3	10.0	3.7	96.4	89.0	59.6	40.4
	2015	26 378	87.3	9.5	3.2	96.6	89.1	59.1	40.9
	2016	27 212	88.4	8.8	2.9	96.8	90.0	59.6	40.4
	2017	27 397	89.0	7.8	3.1	97.1	91.8	62.5	37.5
	2018	26 531	89.4	7.1	3.5	97.3	92.8	64.1	35.9
	2019	26 582	89.7	6.8	3.6	97.3	94.1	66.4	33.6
	2020	26 614	89.6	7.2	3.1	97.4	94.7	67.7	32.3
	2021	26 250	90.6	6.1	3.3	97.4	95.2	70.9	29.1
Gall bladder	2012	122 513	57.5	19.6	22.9	92.1	62.8	26.3	73.7
	2013	129 162	69.9	24.1	5.9	92.2	65.4	27.3	72.7
	2014	131 182	70.3	23.3	6.4	92.3	67.4	28.1	71.9
	2015	133 126	70.8	22.8	6.4	92.9	68.4	28.1	71.9
	2016	137 360	72.4	21.3	6.3	93.5	69.4	28.9	71.1
	2017	138 267	72.6	20.1	7.3	93.7	71.4	29.9	70.1
	2018	139 844	72.5	20.1	7.4	94.1	73.1	31.1	68.9
	2019	140 214	73.5	19.4	7.1	94.4	75.7	32.3	67.7
	2020	134 332	72.9	20.2	6.9	94.8	77.7	33.8	66.2
	2021	136 111	73.4	19.7	6.9	95.1	80.5	36.1	63.9
Pancreas	2012	15 550	72.8	8.7	18.5	96.3	86.5	59.9	40.1
	2013	16 380	86.5	11.0	2.4	95.9	87.6	60.2	39.8
	2014	17 313	86.9	9.9	3.3	96.2	89.1	61.3	38.7
	2015	17 407	88.4	9.1	2.4	96.4	90.3	61.6	38.4
	2016	18 238	89.8	8.0	2.3	96.8	91.1	62.4	37.6
	2017	19 138	90.4	7.1	2.5	97.2	92.3	63.9	36.1
	2018	19 152	91.3	6.4	2.3	97.3	93.4	66.5	33.5
	2019	19 703	91.9	6.2	1.9	97.2	95.1	69.2	30.8
	2020	19 947	91.9	6.3	1.8	97.6	95.7	70.4	29.6
	2021	19 722	92.8	5.4	1.8	97.9	96.8	75.3	24.7
Spleen	2012	4142	70.5	9.5	20.0	81.7	75.8	44.4	55.6
	2013	4509	83.2	13.8	3.0	95.2	75.4	43.3	56.7
	2014	4272	85.4	11.5	3.1	94.6	77.5	45.2	54.8
	2015	3568	85.6	12.3	2.1	94.8	78.9	45.5	54.5
	2016	3171	86.8	10.1	3.1	95.7	80.5	48.0	52.0
	2017	2864	87.4	9.3	3.3	95.3	82.3	49.1	50.9
	2018	2544	86.9	9.7	3.4	95.3	84.7	49.3	50.7
	2019	2413	88.1	8.7	3.2	96.2	86.8	54.0	46.0
	2020	2096	88.6	9.2	2.2	96.5	88.3	55.7	44.3
	2021	1833	88.3	8.8	2.8	96.3	90.3	60.3	39.7
Others	2012	28 779	65.7	15.2	19.1	91.0	61.1	27.6	72.4
	2013	36 363	76.1	19.3	4.6	91.5	63.4	28.5	71.5
	2014	39 854	76.6	18.2	5.1	91.9	64.9	29.7	70.3
	2015	41 465	78.0	17.2	4.8	92.4	65.6	29.4	70.6
	2016	43 523	79.4	15.8	4.8	92.7	67.3	30.3	69.7
	2017	45 622	80.1	14.8	5.1	93.1	69.7	32.3	67.7
	2018	46 587	80.2	14.2	5.7	93.8	71.2	33.1	66.9
	2019	50 525	80.9	13.9	5.3	94.3	74.0	35.2	64.8
	2020	50 048	80.4	14.7	4.9	94.3	76.1	37.0	63.0
	2021	51 216	82.1	13.4	4.5	94.9	79.6	39.5	60.5

Table 5 presents the temporal changes in the complication and mortality rates. Operative mortality decreased across all areas compared to a decade ago, with the most significant reductions observed in surgeries involving the pancreas (−46.4%), esophagus (−41.7%), and liver (−34.8%). However, the surgical complications slowly increased or remained stable. A modest decline was observed after experiencing minor peaks over the past 2–3 years.

**TABLE 5:** Annual changes in surgeries of each organ by complication and mortality rates.

Organ	Year	No. of surgeries	No. of postoperative complications (%)^a^	No. of postoperative 30‐day mortalities (%)	No. of operative mortalities (%)^b^
Esophagus	2012	8819	1653 (18.7)	117 (1.3)	315 (3.6)
	2013	8642	1593 (18.4)	121 (1.4)	327 (3.8)
	2014	9021	1679 (18.6)	115 (1.3)	289 (3.2)
	2015	8943	1709 (19.1)	103 (1.2)	304 (3.4)
	2016	9212	1805 (19.6)	100 (1.1)	238 (2.6)
	2017	9359	1938 (20.7)	108 (1.2)	208 (2.2)
	2018	9286	2065 (22.2)	108 (1.2)	246 (2.6)
	2019	9224	2035 (22.1)	119 (1.3)	246 (2.7)
	2020	8713	1963 (22.5)	95 (1.1)	212 (2.4)
	2021	8905	2011 (22.6)	84 (0.9)	183 (2.1)
Stomach and duodenum	2012	76 186	6447 (8.5)	1085 (1.4)	2381 (3.1)
	2013	75 583	6380 (8.4)	1059 (1.4)	2269 (3.0)
	2014	74 920	6328 (8.4)	1064 (1.4)	2174 (2.9)
	2015	73 877	6418 (8.7)	1007 (1.4)	2110 (2.9)
	2016	72 234	6413 (8.9)	1066 (1.5)	2016 (2.8)
	2017	68 287	6455 (9.5)	1046 (1.5)	1863 (2.7)
	2018	65 152	6228 (9.6)	1048 (1.6)	1833 (2.8)
	2019	63 610	6159 (9.7)	1022 (1.6)	1826 (2.9)
	2020	57 171	5849 (10.2)	977 (1.7)	1679 (2.9)
	2021	56 759	5741 (10.1)	1047 (1.8)	1612 (2.8)
Small intestine and colon	2012	184 810	15 395 (8.3)	3564 (1.9)	6583 (3.6)
	2013	198 677	16 709 (8.4)	3723 (1.9)	6803 (3.4)
	2014	206 857	17 776 (8.6)	3822 (1.9)	6961 (3.4)
	2015	214 453	18 372 (8.6)	4019 (1.9)	7092 (3.3)
	2016	218 228	19 020 (8.7)	3933 (1.8)	6621 (3.0)
	2017	235 359	21 854 (9.3)	4588 (1.9)	7118 (3.0)
	2018	236 496	21 881 (9.3)	4452 (1.9)	7116 (3.0)
	2019	239 612	22 061 (9.2)	4671 (1.9)	7298 (3.0)
	2020	238 631	22 344 (9.4)	4791 (2.0)	7261 (3.0)
	2021	240 448	21 581 (9.0)	4609 (1.9)	6730 (2.8)
Rectum and anus	2012	49 704	4488 (9.0)	462 (0.9)	802 (1.6)
	2013	49 980	4684 (9.4)	517 (1.0)	858 (1.7)
	2014	51 454	4711 (9.2)	449 (0.9)	792 (1.5)
	2015	56 092	4986 (8.9)	519 (0.9)	824 (1.5)
	2016	55 666	5194 (9.3)	503 (0.9)	766 (1.4)
	2017	56 144	5600 (10.0)	556 (1.0)	829 (1.5)
	2018	56 162	5622 (10.0)	522 (0.9)	803 (1.4)
	2019	57 706	5573 (9.7)	563 (1.0)	839 (1.5)
	2020	55 536	5383 (9.7)	555 (1.0)	797 (1.4)
	2021	56 536	5250 (9.3)	577 (1.0)	798 (1.4)
Liver	2012	26 288	2454 (9.3)	310 (1.2)	605 (2.3)
	2013	25 814	2549 (9.9)	275 (1.1)	575 (2.2)
	2014	26 518	2466 (9.3)	246 (0.9)	481 (1.8)
	2015	26 378	2537 (9.6)	234 (0.9)	451 (1.7)
	2016	27 212	2543 (9.3)	222 (0.8)	382 (1.4)
	2017	27 397	2724 (9.9)	214 (0.8)	364 (1.3)
	2018	26 531	2737 (10.3)	189 (0.7)	372 (1.4)
	2019	26 582	2624 (9.9)	201 (0.8)	334 (1.3)
	2020	26 614	2804 (10.5)	338 (1.3)	475 (1.8)
	2021	26 250	2573 (9.8)	271 (1.0)	390 (1.5)
Gall bladder	2012	122 513	4587 (3.7)	531 (0.4)	1082 (0.9)
	2013	129 162	4982 (3.9)	546 (0.4)	1130 (0.9)
	2014	131 182	5020 (3.8)	569 (0.4)	1097 (0.8)
	2015	133 126	5231 (3.9)	541 (0.4)	1036 (0.8)
	2016	137 360	5320 (3.9)	559 (0.4)	980 (0.7)
	2017	138 267	5761 (4.2)	576 (0.4)	968 (0.7)
	2018	139 844	5964 (4.3)	584 (0.4)	954 (0.7)
	2019	140 214	5748 (4.1)	565 (0.4)	935 (0.7)
	2020	134 332	5888 (4.4)	620 (0.5)	978 (0.7)
	2021	136 111	5702 (4.2)	612 (0.4)	930 (0.7)
Pancreas	2012	15 550	2595 (16.7)	213 (1.4)	437 (2.8)
	2013	16 380	2917 (17.8)	211 (1.3)	482 (2.9)
	**2014**	17 313	2966 (17.1)	195 (1.1)	423 (2.4)
	2015	17 407	3229 (18.6)	185 (1.1)	379 (2.2)
	2016	18 238	3543 (19.4)	185 (1.0)	390 (2.1)
	2017	19 138	4076 (21.3)	219 (1.1)	365 (1.9)
	2018	19 152	4309 (22.5)	178 (0.9)	325 (1.7)
	2019	19 703	4522 (23.0)	199 (1.0)	335 (1.7)
	2020	19 947	4520 (22.7)	205 (1.0)	345 (1.7)
	2021	19 722	4415 (22.4)	164 (0.8)	290 (1.5)
Spleen	2012	4142	528 (12.7)	84 (2.0)	138 (3.3)
	2013	4509	575 (12.8)	79 (1.8)	139 (3.1)
	2014	4272	549 (12.9)	88 (2.1)	137 (3.2)
	2015	3568	543 (15.2)	88 (2.5)	144 (4.0)
	2016	3171	449 (14.2)	76 (2.4)	117 (3.7)
	2017	2864	434 (15.2)	65 (2.3)	89 (3.1)
	2018	2544	418 (16.4)	69 (2.7)	104 (4.1)
	2019	2413	380 (15.7)	71 (2.9)	97 (4.0)
	2020	2096	313 (14.9)	63 (3.0)	87 (4.2)
	2021	1833	270 (14.7)	49 (2.7)	69 (3.8)
Others	2012	28 779	4388 (15.2)	1399 (4.9)	2293 (8.0)
	2013	36 363	4712 (13.0)	1401 (3.9)	2346 (6.5)
	2014	39 854	5176 (13.0)	1521 (3.8)	2489 (6.2)
	2015	41 465	5380 (13.0)	1541 (3.7)	2545 (6.1)
	2016	43 523	5975 (13.7)	1760 (4.0)	2684 (6.2)
	2017	45 622	6539 (14.3)	1909 (4.2)	2699 (5.9)
	2018	46 587	6645 (14.3)	1865 (4.0)	2710 (5.8)
	2019	50 525	7750 (15.3)	2221 (4.4)	3220 (6.4)
	2020	50 048	7838 (15.7)	2267 (4.5)	3284 (6.6)
	2021	51 216	7697 (15.0)	2293 (4.5)	3173 (6.2)

^a^ Complications were defined by Clavien–Dindo grade IIIa–V.

^b^ Operative mortality was a rate that combined 30‐day mortality and hospitalization death in 31–90 days after surgery.

### 3.3 Endoscopic surgery rate in major surgical procedures

**FIGURE 2:**
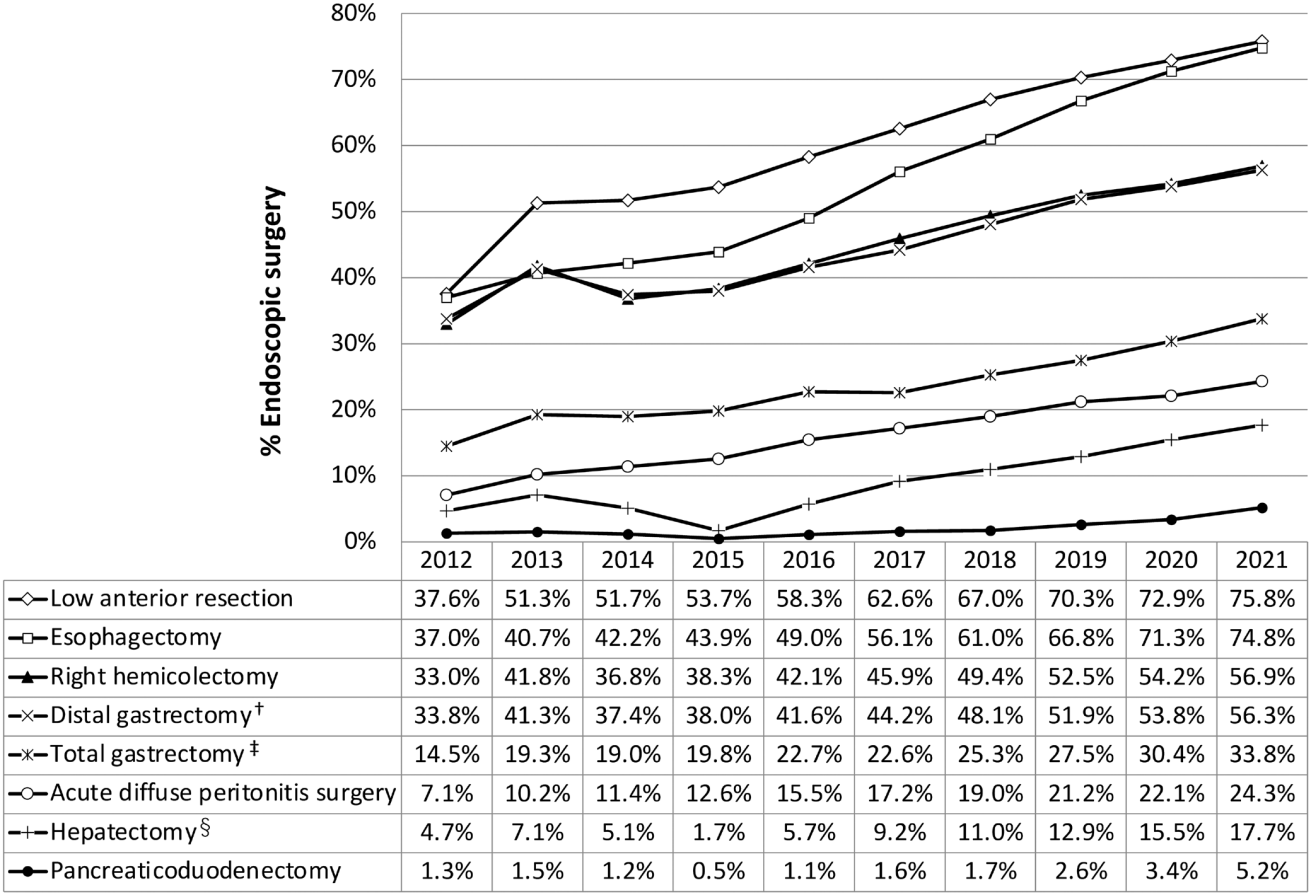
Annual changes in the rate of endoscopic surgery in the eight major surgical procedures. † including pylorus‐preserving gastrectomy and segmental gastrectomy, ‡ including proximal gastrectomy, § segmentectomy or more; excluding lateral segmentectomy.

Figure 2 presents the trends in the endoscopic surgery rates across eight major surgical procedures. Within these procedures, established as benchmarks for improving surgical quality, the rates of endoscopic surgery have maintained their trend in recent years, still increasing by approximately 3% annually. Particularly, esophagectomy has exhibited the most significant increase in endoscopic surgery since 2016.

### 3.4 Operative procedures with high rates of emergency surgeries

Table 6 lists the top 20 procedures with the highest rates of emergency surgery among those performed in more than 100 cases annually, out of 80 applicable procedures. The list predominantly includes procedures related to abdominal trauma, acute abdomen, and oncology emergencies. Compared to the remaining 60 procedures, these 20 procedures have a lower participation rate of board‐certified surgeons (median, 80.0% vs. 90.9%), and notably higher rates of postoperative complications (18.7% vs. 12.0%), re‐operations (7.8% vs. 3.9%), postoperative 30‐day mortalities (4.5% vs. 0.9%), and operative mortalities (7.2% vs. 1.4%).

**TABLE 6:** Top 20 operative procedures with the highest rates of emergency surgery.

Order	Organ/difficulty level	Operative procedure	No. of surgeries	Emergency surgeries (%)	Board‐certified surgeon participation (%)	Postoperative complications (%)^a^	Re‐operations (%)	Postoperative 30‐day mortalities (%)	Operative mortalities (%)^b^
1	Ot/med	Acute diffuse peritonitis surgery	15 542	92.6	80.3	27.3	7.8	8.0	11.5
2	St/low	Gastric suture^c^	5327	91.5	75.1	17.7	5.7	5.7	7.5
3	Ot/med	Gastrointestinal perforation closure	421	90.5	80.3	35.4	14.0	11.6	16.2
4	Li/high	Surgery for hepatic trauma^d^	394	82.0	58.6	48.5	49.0	22.8	26.6
5	In/low	Disinvagination (invasive)	152	80.3	76.3	5.3	2.0	2.6	3.3
6	Es/med	Esophageal suture (perforation, injury)	183	74.9	86.9	36.6	13.7	3.3	4.9
7	Ot/low	Localized intra‐abdominal abscess surgery	2376	70.5	75.8	14.6	6.6	2.2	3.2
8	In/med	Intestinal obstruction surgery (with enterectomy)	26 275	68.3	76.6	10.2	4.4	2.4	3.4
9	In/low	Appendectomy	56 071	67.6	68.3	1.8	1.0	0.1	0.2
10	In/low	Partial small bowel resection (benign)	9017	62.5	78.9	20.4	10.4	6.7	9.1
11	Re/med	Hartmann's procedure	6408	58.6	83.1	20.8	6.0	5.6	7.5
12	Gb/high	Surgery for bile duct trauma^e^	235	52.8	90.2	27.2	11.1	5.1	6.8
13	Gb/low	External cholecystostomy	139	51.8	65.5	24.5	14.4	4.3	10.1
14	In/low	Ileocecal resection (benign)	4970	49.1	79.0	10.3	4.1	2.2	3.0
15	In/low	Partial colectomy and sigmoid colectomy (benign)	8536	45.3	80.8	15.5	6.3	4.1	5.6
16	St/low	Gastric pyloroplasty	106	43.4	57.5	4.7	5.7	0.9	0.9
17	Ot/med	Diaphragm suture	294	40.5	83.2	19.7	9.5	4.4	6.5
18	In/med	Total colectomy	1546	33.4	84.0	23.9	9.5	7.9	9.8
19	Ot/low	Exploratory laparotomy	12 401	30.3	82.5	16.8	13.2	6.2	9.0
20	In/low	Enterotomy and enterorrhaphy	4268	29.7	78.9	17.0	7.7	4.5	7.5

Abbreviations: Es, esophagus; Gb, gall bladder; In, small intestine and colon; Li, liver; Ot, others; Re, rectum; St, stomach and duodenum.

^a^ Complications were defined by Clavien–Dindo grade IIIa–V.

^b^ Operative mortality was a rate that combined 30‐day mortality and hospitalization death in 31 to 90 days after surgery.

^c^ Gastric suture includes gastric suture for gastric rupture, suture closure for gastroduodenal perforation, and omental implantation and omental transposition.

^d^ Surgery for hepatic trauma excludes drainage only.

^e^ Surgery for bile duct trauma excludes drainage only.

## 4 DISCUSSION

By comprehensively reviewing data from actual clinical practices in Japan registered in the NCD, this section addresses the following: the number of surgeries, patient demographics, surgical environments, endoscopic surgery, and surgical outcomes, particularly for the esophagus, liver, pancreas, and Acute Care Surgery (ACS), and highlights studies focusing on the gender of surgeons.

The impact of the coronavirus disease 2019 pandemic has led to a decrease in surgeries across all areas,^28^ with an overall reduction of 2.7%.^26^ However, the data suggest a return to the original trends by 2021. This result indicates that surgical care was gradually returning to normalcy as treatments and the healthcare system for the coronavirus disease 2019 were organized, clarifying appropriate measures for surgical patients. Upon closer examination, it appears that the increase in surgical procedures in the small intestine, colon, rectum and anus, gallbladder, pancreas, and others may be attributed to improved diagnostic capabilities for diseases and the growing older adult population. Conversely, the decrease in the number of stomach and duodenum, and spleen surgeries may be influenced by a reduction in disease incidence rates and the development of alternative treatment methods.^29^, ^30^

Consistent with earlier reports, there was a clear trend towards aging in patients undergoing surgery. Notably, there has been a significant increase in the proportion of patients ≥80 years, which is likely attributable to the rise in the aging population^31^ and the extension of healthy life expectancy.^32^ However, the proportion of patients <60 years undergoing surgery for rectal and anal issues has exhibited a slight increase, likely due to the westernization of diets, stress, and decreased physical activity, contributing to the incidence of rectal tumors.^33^ While the proportion of male patients remained high in all areas, the last decade has generally demonstrated a shift towards an increasing ratio of female patients. This trend can be attributed to the growing aging population and the higher proportion of women in the older adult population.^31^

The trend of surgeries being conducted in certified institutions under the management of anesthesiologists, with the involvement of board‐certified surgeons, is becoming consistently stronger despite some variations across different organs. Receiving surgical training in a well‐equipped environment is beneficial for trainee surgeons, and most importantly, maintaining a high surgical standard is advantageous and desirable for patients.^34^

The trend of increasing endoscopic surgery is expected to persist in the foreseeable future. This trend has been significantly influenced by the widespread adoption of robotic surgery into daily practice, in addition to laparoscopic and thoracoscopic surgeries. As the advantages of robotic surgery become increasingly apparent, particularly in procedures where endoscopic surgery is relatively less common, the proportion of endoscopic surgeries is likely to further increase. Moreover, there is considerable anticipation regarding the performance and innovative features of surgical robots that will emerge in the future.

General trends in surgical outcomes remained consistent. The most significant observation was the uniform decline in operative mortality across all areas. There is substantial significance in maintaining these results as the number of surgeries and the proportion of older adult patients continue to increase. This reflects the tangible efforts of Japanese gastrointestinal surgeons to improve outcomes. However, an increase in complications, diverging from the trend in operative mortality, has been noted previously, making the control of complications an ongoing challenge.

We further focus on the surgical outcomes of two critical issues: the first concerns surgeries involving the esophagus, liver, and pancreas, and the second pertains to ACS. The esophagus, liver, and pancreas share the common outcome of substantial improvements in operative mortality in recent years. A distinguishing characteristic is the limited number of surgeries compared to other areas, with a relatively high proportion of these being high‐difficulty surgeries. However, what appears most crucial is the notably high participation rate of board‐certified surgeons, which can be interpreted as contributing to favorable outcomes. While the importance of board‐certified surgeons in surgeries has already been reported in some quarters,^35^, ^36^ the matter at hand involves devising ways to train more board‐certified surgeons and enhance their engagement across a broader range of organ specialties.

The second issue is how to improve ACS outcomes. The traditional pillars of ACS encompass trauma, emergency general surgery, and surgical critical care,^37^ with surgical rescue being recently added to these core areas.^38^ These are characterized by a lower participation rate of board‐certified surgeons compared to other procedures, as well as higher rates of complications, re‐operations, and notably worse morbidities. Unlike elective surgeries that pursue curative treatment from an anatomical perspective, the formulation of treatment strategies for time‐sensitive surgeries requires a different approach. Merely increasing the participation rate of board‐certified surgeons may not be sufficient to improve ACS outcomes significantly. To improve surgical outcomes for severe traumas, including hepatic trauma which exhibited the highest operative mortality in this data set, it may be necessary to establish specialized institutions, such as trauma centers and ACS centers, to a certain extent, and consolidate cases similar to practices in Western countries.^39^

Enhancing the education of gastroenterological surgeons in the field of ACS and strengthening their collaboration with acute care surgeons could further accelerate the development of gastroenterological surgery. Notably, the Japanese ACS Society became an associate member of the NCD in 2021, suggesting the establishment of a cooperative environment in both clinical and research aspects.

Between 2013 and 2022, 98 research projects in the field of gastroenterological surgery that use big data from the NCD were approved and reported on a rolling basis in scholarly papers. Among them, 12 papers were published in 2022, covering topics such as high‐complexity esophageal^40^, ^41^, ^42^ and pancreatic^43^, ^44^ cancer surgery, coronavirus disease 2019‐related issues,^45^, ^46^ and surgeon's sex‐related aspects,^47^, ^48^ which indicates a heightened interest in these areas.

Given that this is the first large‐scale study on the surgeon's sex in the field of gastroenterological surgery, an overview is provided here. One of the two studies compared the surgical experience available to surgeons of different sexes, while the other compared surgical outcomes based on the sex of gastroenterological surgeons. The first study found that female surgeons had less surgical experience than male surgeons in Japan, and this gap tended to widen with an increase in years of experience, especially for medium‐ and high‐difficulty procedures.^47^ The second study found that female gastrointestinal surgeons were more frequently responsible for patients with comorbid conditions, although they performed fewer surgeries than their male counterparts. Despite these disadvantages, no significant differences were observed in the risk of surgical mortality between male and female surgeons.^48^ Both studies yield important conclusions that suggest addressing these gender gaps could further advance the field of gastroenterological surgery.

In summary, we presented the short‐term outcomes of 2021 and the surgical trends of the decade based on the NCD, along with the latest research findings. It is evident that the NCD will continue to be a critical cornerstone for the future development of gastroenterological surgery.

## AUTHOR CONTRIBUTIONS

**Sunao Ito:** Conceptualization; project administration; writing – original draft. **Arata Takahashi:** Data curation; investigation; methodology; resources; software. **Hideki Ueno:** Conceptualization; project administration; supervision; writing – review and editing. **Shuji Takiguchi:** Conceptualization; project administration; writing – original draft. **Yoshiki Kajiwara:** Conceptualization; data curation; formal analysis; project administration; writing – original draft; writing – review and editing. **Yoshihiro Kakeji:** Conceptualization; supervision; writing – review and editing. **Susumu Eguchi:** Conceptualization; supervision; writing – review and editing. **Takanori Goi:** Conceptualization; supervision; writing – review and editing. **Akio Saiura:** Conceptualization; supervision; writing – review and editing. **Akira Sasaki:** Conceptualization; supervision; writing – review and editing. **Hiroya Takeuchi:** Conceptualization; supervision; writing – review and editing. **Chie Tanaka:** Conceptualization; supervision; writing – review and editing. **Masaji Hashimoto:** Conceptualization; supervision; writing – review and editing. **Naoki Hiki:** Conceptualization; supervision; writing – review and editing. **Akihiko Horiguchi:** Conceptualization; supervision; writing – review and editing. **Satoru Matsuda:** Conceptualization; supervision; writing – review and editing. **Tsunekazu Mizushima:** Conceptualization; supervision; writing – review and editing. **Hiroyuki Yamamoto:** Conceptualization; data curation; formal analysis; investigation; methodology; resources; supervision; writing – review and editing. **Yuko Kitagawa:** Conceptualization; project administration; supervision; writing – review and editing. **Ken Shirabe:** Conceptualization; project administration; supervision; writing – review and editing.

## CONFLICT OF INTEREST STATEMENT

Yuko Kitagawa is Editor‐in‐Chief and Hideki Ueno, Hiroya Takeuchi, Naoki Hiki, and Akihiko Horiguchi are Associate Editors of *Annals of Gastroenterological Surgery*. Shuji Takiguchi, Yoshihiro Kakeji, Susumu Eguchi, Takanori Goi, Akio Saiura, Chie Tanaka, Satoru Matsuda, Tsunekazu Mizushima, and Ken Shirabe are editorial board members of *Annals of Gastroenterological Surgery*. Arata Takahashi and Hiroyuki Yamamoto are affiliated with the Department of Healthcare Quality Assessment at the University of Tokyo that is a social collaboration department supported by grants from the National Clinical Database, Johnson & Johnson K.K., and Nipro Co and Intuitive Surgical Sàrl. Other authors have no conflicts of interest.

## ETHICS STATEMENT

Approval of the research protocol by an Institutional Reviewer Board: N/A.

Informed Consent: N/A.

Registry and the Registration No. of the study/trial: N/A.

Animal Studies: N/A.
